# Riparian spiders make pyriform silk attachment discs that stick better when wet than those of terrestrial spiders

**DOI:** 10.1242/jeb.250902

**Published:** 2025-11-10

**Authors:** Bernd F. Steklis, Todd A. Blackledge

**Affiliations:** Department of Biology and Integrated Bioscience Program, University of Akron, Akron, OH 44325, USA

**Keywords:** Silk protein, Wet adhesion, Spider silk adhesion

## Abstract

Adhesion in wet conditions presents significant challenges due to the disruptive effects of water on interfacial bonding, spreading and curing. Many organisms have evolved adhesives that adhere strongly in damp or submerged environments. However, the pyriform silk attachment discs of the terrestrial western black widow spider lose ∼8 times their adhesive strength when wet. Here, we tested the hypothesis that riparian species of spiders have evolved attachment discs that are resistant to the adverse effects of water on adhesion. We compared adhesion of attachment discs from three terrestrial spiders from relatively dry habitats with those of three riparian spider species when discs were loaded under both dry and wet conditions. Failure modes shifted from dragline breakage in dry conditions to adhesive failure in wet conditions across all species, highlighting the impact of water on interfacial bonding. However, riparian species’ attachment discs maintained adhesive force when wet while terrestrial species experienced ∼50% reduction in peak force and work of adhesion in wet conditions. These findings suggest that riparian spider silks have evolved specializations that maintain adhesive performance of pyriform attachment discs in wet environments, offering insights into bioinspired design for water-resistant adhesives.

## Teaser

**Summary:** Attachment discs of riparian spiders maintain adhesion in wet conditions, highlighting ecological adaptation and informing biomimetic design of water-resistant adhesives.

## INTRODUCTION

Water is a fundamental problem for adhesion in nature. Both functional morphology and bioinspired studies often focus on fully aquatic organisms such as sandcastle worms, mussels and caddisflies (Fant et al., 2010; Narayanan et al., 2021; Stewart and Wang, 2010). However, water is also a challenge for adhesion in terrestrial ecosystems. A wet surface reduces the contact area of an applied adhesive and alters the surface energy of the substrate, affecting an adhesive's ability to spread (Narayanan et al., 2021). Additionally, water can compete with an adhesive's functional groups for interactions with the substrate, significantly decreasing the overall adhesive strength of the system (Cui and Liu, 2021; Narayanan et al., 2021). Water may also slow down the curing process, reducing cohesive strength. Even after application, water can degrade an adhesive through hydrolysis, swelling or fouling (Mao et al., 2020; Wang et al., 2023). Wet surfaces can also decrease viscosity, reducing the cohesive strength of the adhesive material (Narayanan et al., 2021). Therefore, it is no surprise that water often impairs adhesive performance in many land-dwelling species. Geckos' toe pad adhesive shear strength decreases significantly when wetted (Stark et al., 2012). Tree frogs' toe pads must remain moist enough to spread sufficiently on the surfaces they traverse but too much water abolishes the meniscus around the edge of the pads, significantly reducing adhesive and frictional forces (Meng et al., 2019). A similar tradeoff is seen in bat species that rely on wet adhesive sucker-feet to cling to surfaces, where the adhesive material relies on moderate humidity to be viscous enough to spread on a surface without losing cohesive properties (Riskin and Racey, 2009). The western black widow (*Latrodectus hesperus*) also shows a significant, ∼8 times, decrease in adhesive force of pyriform silk attachment discs when wet compared with dry (Steklis et al., 2025). These examples suggest that water should act as a selective force on adhesive performance in terrestrial adhesive systems.

Spiders are an excellent subject for adhesive studies because there are over 53,157 species with diverse ecologies, many of which live in environmental conditions that are challenging for adhesion (World Spider Catalog, 2025). Spiders also have a variety of adhesive systems that sometimes leverage water for improved performance. For instance, *Cyrtarachne akirai* produces specialized silk capture threads that are over-saturated with water, allowing the glue to spread quickly on moth wings but then rapidly harden (Diaz et al., 2018). Viscid glues of orb-weaving spiders are hygroscopic and promote adhesion by tuning viscosity to optimize the trade-off between spreading and cohesion (Amarpuri et al., 2015). In contrast, the same type of glue produced by a cobweb spider is largely invariant to humidity (Sahni et al., 2011). Spiders also modulate the morphological and chemical composition of various silks in response to challenging environmental conditions such as high wind or high humidity (Opell et al., 2018; Wu et al., 2013). Some species from heavy rainfall environments have stronger and tougher major ampullate silk in comparison to that of species from drier environments, perhaps minimizing damage from frequent rainfall (Hopfe et al., 2024). These examples highlight the adaptive flexibility of spider silks to environmental conditions; however, few studies have examined spider attachment discs in this context (Grawe et al., 2014; Steklis et al., 2025).

Attachment discs are made of pyriform silk and secure trailing safety draglines or anchor webs to the environment. Attachment discs are spun in fractions of a second, adhere strongly enough to withstand the force of the spider's body weight falling, and maintain adhesion for long periods of time in between web repairs (Wolff et al., 2015). Attachment discs are composed of silk fibers embedded in a gluey matrix, spun over or around the dragline threads that they secure. The fibrous and gluey components of the pyriform silk are extruded simultaneously from the same silk gland (Kovoor and Zylberberg, 1982; Wolff et al., 2015). These discs consist of three primary regions: the baseplate, bridge and conjunction (Grawe et al., 2014). The baseplate directly contacts the surface and serves as the main adhesive element of the disc (Wolff et al., 2015). The bridge is formed by overlapping silk fibers coated in glue, connecting the baseplate to the conjunction, which in turn secures the dragline fiber to the disc. The glue in the baseplate is the only material in contact with the substrate and provides the main adhesive strength, while the other regions contribute to the cohesive strength of the overall structure (Wolff et al., 2015). While the baseplate glue appears to be a solidified liquid, it is actually a matrix of nanofibrils, crosslinked proteins and lipids (Wolff et al., 2015). This gluey matrix accounts for less than half of the total secretion and is initially extruded as a liquid that solidifies within milliseconds, producing small water droplets in the process (Wolff et al., 2015). The solidification process is thought to involve a peptide bonding condensation reaction, which releases water droplets during curing. This suggests that the adhesive cure may be reversible in certain wet conditions, reducing adhesive force. The baseplate of the attachment disc is also extremely thin, allowing any solvents or water molecules produced during curing to evaporate quickly, resulting in rapid curing times. These processes, however, may be influenced by the presence of water. For instance, the adhesion of western black widow attachment discs is about 8 times lower when loaded in wet conditions compared with dry (Steklis et al., 2025). Terrestrial organisms also face a distinct challenge compared with aquatic organisms in that the environment in which an adhesive is applied can differ from the conditions during subsequent loading of adhesives. For example, spiders may build a web in initially dry conditions, but the web must maintain adhesion when it rains or as humidity increases. In the case of black widows, adhesion of pyriform discs is robust to the presence of water during spinning and placement as long as the discs are dry when loaded (Steklis et al., 2025). Almost all spiders live on land except for the fully aquatic *Argyroneta* spp.; however, here we use ‘terrestrial’ to denote species that live on land in low humidity or low water presence environments compared with riparian specialists that are in habitats with significantly higher humidity and often have surfaces covered by water. Here, we tested the hypothesis that riparian species of spiders have evolved attachment discs that are more resistant to the negative impacts of water on adhesive performance compared with those of terrestrial species.

## MATERIALS AND METHODS

### Spiders

This study includes three terrestrial and three riparian spiders from phylogenetically diverse species to compare independent transitions between ecologies. The three riparian species are associated with water in different ways. The six-spotted fishing spider, *Dolomedes triton* (Walckenaer 1837) (Dolomedidae), is a wandering hunter that moves across water surfaces hunting for prey, often trailing a dragline that contacts the water. *Dolomedes triton* mostly places attachment discs on riparian plants and substrates near water for traversal or retreat construction. *Tetragnatha elongata* Walckenaer 1841 (Tetragnathidae) builds orb webs in close proximity to ponds and streams. Its attachment discs therefore must hold webs securely under high humidity. *Wendilgarda clara* Keyserling 1886 (Theridiosomatidae) is a small ray spider that builds modified orb webs above streams in rainforests. The frames of the webs are attached to nearby substrates, but the glue-coated capture lines adhere directly to the water surface (Coddington and Valerio, 1980). Here, we focused solely on pyriform discs used to secure dragline silk to substrates. Thus, each riparian species produces attachment discs that are routinely placed and subsequently loaded in wet conditions.

The three terrestrial species inhabit variable, but often dry, environments. The western black widow, *Latrodectus hesperus* Chamberlin & Ivie 1935 (Theridiidae), builds cobwebs in sheltered locations and shows reduced adhesion in wet conditions (Steklis et al., 2025). The furrow orb spider, *Larinioides cornutus* (Clerck 1757), builds webs primarily in shrubs and grasses in relatively mesic areas. While *L. cornutus* habitat broadly overlaps with that of *T. elongata*, *L. cornutus* tends to build webs much further away from water. *Larinioides cornutus* was also found to have a lower optimal humidity for its viscid capture silk adhesion (∼50% relative humidity, RH) compared with *Tetragnatha laboriosa* (>90% RH), a species ecologically similar to *T. elongata* (Opell et al., 2018). This suggests that *L. cornutus*' foraging habitat is relatively dry compared with that of *T. laboriosa* and potentially *T. elongata* as well. The Pennsylvania grass spider, *Agelenopsis pennsylvanica* (C. L. Koch 1843) (Agelenidae), builds funnel webs around human-made structures, grasslands and forests. However, its webs are rarely built on wet substrates. The three terrestrial species are predicted to show reduced adhesion in wet loading conditions compared with dry, while the three riparian species are predicted to show no change in performance across wet and dry conditions if their attachment discs evolved specializations to wet environments.

### Spider housing

Eight individual spiders of each species were housed in the lab. *Wendilgarda clara* were housed in 100×80×60 cm plastic terrariums inverted over circular wire frames in shallow bowls of water. The spiders were fed wingless fruit flies (*Drosophila melanogaster*) every other day. *Dolomedes triton* were housed in the same sized plastic terrariums containing a layer of soil, a small paper retreat and a Petri dish filled with water. The spiders were fed crickets (*Gryllodes sigillatus*) every other day. *Tetragnatha elongata* were housed in large glass jars (9×12 cm) with a paper inner wall and a stick. The spiders were lightly misted with water but did not build webs and were only held for 3–4 days, just long enough to collect their attachment discs. *Larinioides cornutus* were housed in 12×40×40 cm frame cages, built orb webs frequently and were fed crickets every other day. *Agelenopsis pennsylvanica* were housed in the same conditions as *D. triton*, but without the Petri dish of water. The *Latrodectus hesperus* data included in this study were obtained from Steklis et al. (2025) and the housing details can be found there. All species besides *L. hesperus* and *W. clara* were collected in Bath Nature Preserve, OH, USA, or on the University of Akron campus, OH, USA. *Latrodectus hepserus* were purchased from bugsofamerica.com ∼7 months prior to the experiment. *Wendilgarda clara* were collected in Puerto Rico by Dr Angela Alicea-Serrano and transported back to the lab in Akron, OH, USA.

### Attachment disc collection

Attachment discs were collected as outlined by Steklis et al. (2025), with minor adjustments. Glass microscope slides were cleaned using 90% ethanol, rinsed thoroughly with deionized water, and then dried. This standardized the potential influence of varying ion concentrations and pH of water on the molecular structure of pyriform silk proteins. Attachment discs were applied to the clean slides in a dry environment (∼40–60% RH). Glass is a relatively smooth and hydrophilic substrate compared with many natural surfaces and therefore does not represent all of the surfaces to which spiders attach their discs (Wolff et al., 2020). However, it provides a highly repeatable substrate for comparison among species.

**Fig. 1.**
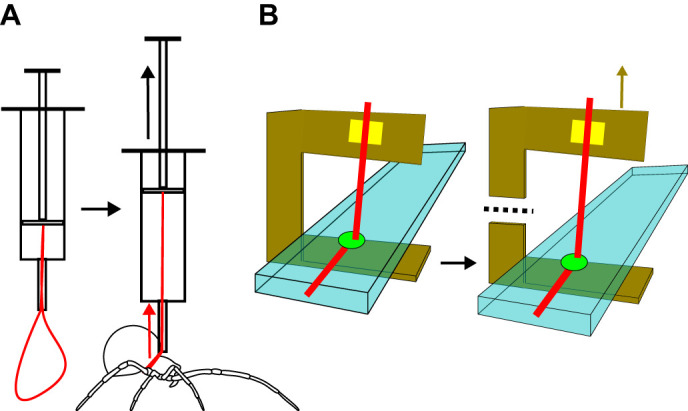
**Sample collection and testing methods.** (A) The ‘spider lasso’ is a loop of nylon thread protruding through a syringe that could be looped around the spider to allow controlled attachment disc placement. (B) An attachment disc sample mounted in a C-shaped cardboard cutout on a glass microscope slide with the attachment disc highlighted in green, the dragline highlighted in red, a piece of double-sided tape securing the dragline to the frame highlighted in yellow, the cut line marked with a dashed line, and the direction the Nano Bionix tensile tester pulls on the ‘C’ frame marked with a brown arrow. Modified from Steklis et al. (2025).

Each slide was first fitted with a cardboard C-shaped cutout, creating a 10 mm gap between the glass surface and the upper edge of the cutout, which was folded perpendicular to the slide. This design enabled the dragline to be secured to the top of the ‘C’ after the attachment discs were placed on the glass (Fig. 1B). All spiders, except *W. clara*, were restrained using a nylon loop ‘lasso’ made from fishing line passed through a syringe, which allowed the loop to tighten around the spider's pedicel (Fig. 1A). This setup permitted the spiders to walk semi-naturally while still controlling the placement location of the attachment discs. The spider was first positioned on the glass slide with its spinnerets directly under the upper edge of the cutout and gently guided until an attachment disc was spun. The spider was then gently lifted towards the upper edge of the cutout, minimizing pull on the dragline. The dragline was then attached to the upper edge of the cutout using double-sided tape, and the trailing end was cut to separate the spider from the sample. A small square of cardboard was placed on the tape to secure the dragline. *Wendilgarda clara* were significantly smaller than other species so the lasso could not be used. Instead, *W. clara* spiders were placed on the glass slide and allowed to walk freely across the surface until an attachment disc was placed. The slide was then flipped over, causing the spider to hang from the dragline and making it possible to install the ‘C’ cutout and secure the dragline in the frame. These discs were observed closely before testing to ensure they were not visibly damaged from the weight of the spider hanging from the dragline before the frame was placed and the dragline was secured.

### Adhesion testing

Adhesion testing followed the procedures described in Steklis et al. (2025). Each attachment disc was imaged under a Leica DMLB 2 Clinical Microscope (Leica microsystems, Wetzlar, Germany) at ×10 magnification using Olympus Q-Color5 imaging system (Olympus, Tokyo, Japan) and recorded using QCapture64 (QImaging, Surrey, BC, Canada) for area and morphology analysis. The sample was then placed in a Nano Bionix tensile tester (MTS System Corp., Oak Ridge, TN, USA), with the lower grip holding the slide and the upper grip holding the cardboard cutout where the dragline was secured. The cardboard ‘C’ was cut near its midpoint, separating the dragline end from the attachment disc end, and the sample was adjusted in the *X*–*Y* plane to ensure that the dragline pulled the attachment disc perpendicular to the slide surface, controlling for the effect of different pulling angles (Sahni et al., 2012). The dragline was pulled at a rate of 0.1 mm s^−1^ until complete failure was observed (Blackledge et al., 2005; Swanson et al., 2006). The *W. clara* samples were pulled at 0.15 mm s^−1^ because of a difference in the Nano Bionix program settings. The force, time and displacement were recorded, and work was calculated. The sample was then placed back under the compound microscope, where images of the attachment disc were taken again to determine the failure type (cohesive, adhesive or dragline). Because we focused on the adhesive properties of the attachment discs themselves, silk tensile properties such as Young's modulus were not included in this study.

Steklis et al. (2025) showed that conditions in which *L. hesperus* attachment discs were loaded influenced performance more than the conditions in which discs were placed. Many spiders are also reluctant to place discs on wet surfaces. Therefore, we chose to vary only the loading conditions of the discs in this study between dry and wet, with all discs initially placed in dry conditions. Dry loading condition tests were performed at room humidity (∼40–60% RH). For the wet loading condition tests, the spider placed an attachment disc on a dry slide, which was then imaged, mounted in the Nano Bionix tensile tester and sprayed with deionized water from an atomizer until the disc was visibly covered by water droplets. All samples were allowed to dry after tensile testing before microscope imaging and failure type analysis.

The *L. hesperus* data were taken from Steklis et al. (2025) and consisted of six attachment discs per individual spider, with three tested in wet conditions and three tested in dry conditions. For all other species, we tested four attachment discs per individual spider – two in wet conditions and two in dry conditions. One *L. hesperus* only produced two discs in wet conditions instead of three and one did not produce any discs for the dry conditions. One *W. clara* produced only one disc in wet conditions instead of two. This resulted in final sample sizes per species of *n*=32 for *T. elongata* (16 dry and 16 wet, eight spiders total), *n*=32 for *L. cornutus* (16 dry and 16 wet, eight spiders total), *n*=31 for *W. clara* (16 dry and 15 wet, eight spiders total), *n*=32 for *A. pennsylvanica* (16 dry and 16 wet, eight spiders total), *n*=40 for *D. triton* (20 dry and 20 wet, ten spiders total) and *n*=50 for *L. hesperus* (27 dry and 23 wet, nine spiders total). A total of 217 attachment discs were tested.

### Failure type/image analysis

**Fig. 2.**
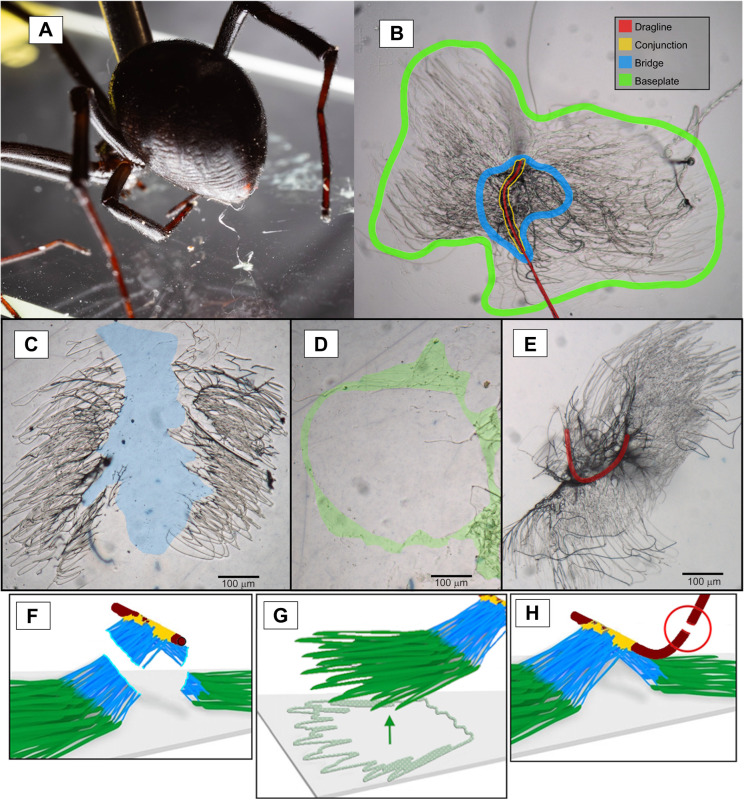
**Attachment disc morphology and failure types of attachment discs.** (A) *Latrodectus hesperus* placing an attachment disc on a glass slide using the spider lasso method. The spider is restrained at the pedicel by a loop of nylon but can move its legs and abdomen naturally. (B) Attachment disc morphology with major components highlighted in color. Only the baseplate (outlined in green) contacts the substrate and is the primary adhesive element with the substrate. The bridge and conjunction (blue and yellow) hold the dragline (red). (C,F) Cohesive failure within the disc (highlighted in blue) left most of the baseplate remaining on the glass. (D,G) Adhesive failure of the complete disc left little to no material along the edge of the baseplate (highlighted in green). (E,H) Dragline failure left the entire attachment disc intact with remaining dragline fragment highlighted in red. Images were taken at ×10 magnification using a compound microscope (Leica DMLB 2 Clinical Microscope). Images were captured using the Olympus Q-Color5 imaging system (Olympus Confocal) and recorded with QCapture64 (QImaging). Scale bars: 100 µm. Modified from Steklis et al. (2025).

**Fig. 3.**
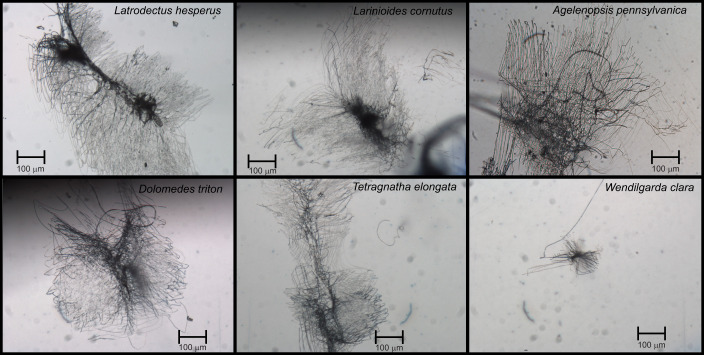
**Images of attachment discs of each species.** Images were taken at ×10 magnification using a compound microscope (Leica DMLB 2 Clinical Microscope). Images were captured using the Olympus Q-Color5 imaging system (Olympus Confocal) and recorded with QCapture64 (QImaging). Scale bars: 100 µm. There were no apparent morphological differences in disc structure between of the species, except for the discs of *Wendilgarda clara*, which were very small because of the small size of the species.

A Leica DMLB 2 Clinical Microscope (Leica Microsystems) at ×10 magnification was used to analyze the attachment disc samples before and after failure. Images were captured using the Olympus Q-Color5 imaging system and recorded using QCapture64 (QImaging). ImageJ (Schneider et al., 2012) was utilized to measure the area of each attachment disc. The program was calibrated with a millimeter scale, and the same scale was used consistently for each measurement. For images of attachment discs post-failure, the freehand selection tool was employed to trace the outer edges of the disc and measure the total area. Any areas where attachment disc material had been removed were also traced and measured. We observed three mechanisms of failure. Dragline failure was classified as the complete attachment disc remaining intact with visible remnants of the ruptured dragline still present. Cohesive failure was classified as more than 40% of the disc remaining adhered to the slide, with clear signs of fracture or tearing visible. Cohesive failure typically presented as a rupture in the conjunction or bridge of the disc, and rarely in the baseplate. Adhesive failure was identified when more than 90% of the disc was removed, leaving minimal traces on the slide. As the baseplate is the only disc region that makes contact with the substrate, adhesive failure was entirely due to the baseplate failing. The amount of disc remaining was highly bimodal so that most samples were unambiguously assignable to either adhesive or cohesive failure. We suspect this is because of a tendency for fracture to occur in the conjunction where piriform secretions are not adhered directly to the substrate. Examples of the observed failure types are shown in Fig. 2, with simplified illustrations. There were slight differences in attachment disc morphology across species but there was no clear difference between habitat groups. Images of attachment discs for each species are shown in Fig. 3.

### Data analysis

A mixed-effects general linear model analyzed the influence of loading condition (wet versus dry) and habitat type (terrestrial versus riparian) on the peak force of adhesion and a second analysis was done using the same model on the work of adhesion. The model included loading condition, habitat type and their interaction (loading habitat×condition type) as fixed effects. Species was treated as a random effect to account for variability among species (especially size differences). Individual spider identity was also included as a random effect to account for repeated measures from individual spiders. The failure type data were analyzed using chi-square tests for each species to compare dry and wet conditions, and also to compare the overall frequencies of failure types between terrestrial and riparian spiders. In samples where the failure type was unclear and exhibited intermediate characteristics between two failure types (*n*=23, ∼10% of discs), two separate datasets were analyzed – one coding all unclear failures in favor of adhesive failure and the other coding them in favor of cohesive failure. Statistical analyses were performed on both datasets to account for the ambiguity in failure classification. All statistics were performed using JMP Pro (version 17.0; SAS Institute Inc., Cary, NC, USA).

## RESULTS

**Fig. 4.**
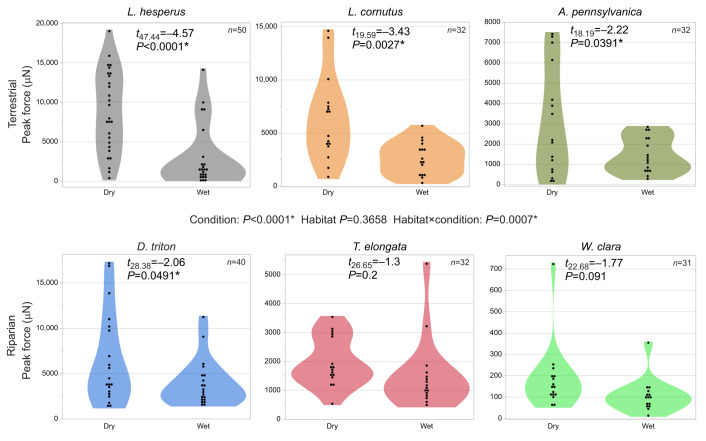
**Peak force of adhesion in dry versus wet conditions.** The top row shows terrestrial species and the bottom row shows riparian species. A mixed-effects general linear model analyzed the influence of loading condition (wet versus dry) and habitat type (terrestrial versus riparian) on the peak force of adhesion. Asterisks indicate significant *P*-values. Two of the three riparian species showed no significant decrease in peak force of adhesion in wet conditions, while there was a marginally significant decrease for *D. triton*. The habitat and condition interaction term was significant, showing that the peak force of species in the riparian habitat group was less affected by wet conditions than that of species in the terrestrial habitat group. Total sample size of discs tested, *N*=217.

**Fig. 5.**
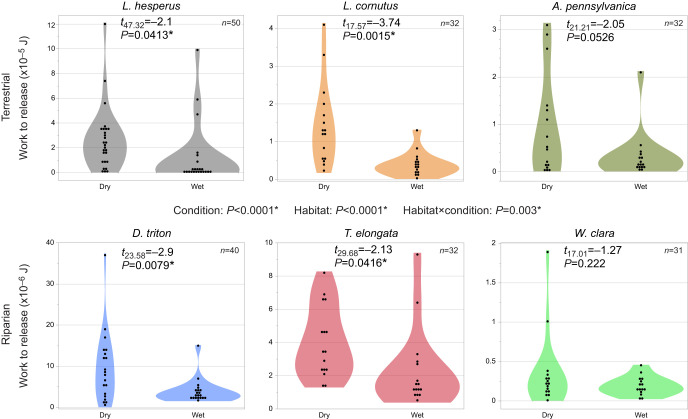
**Work of adhesion for each species in dry and wet conditions.** The top row shows terrestrial species and the bottom row shows riparian species. A mixed-effects general linear model analyzed the influence of loading condition (wet versus dry) and habitat type (terrestrial versus riparian) on the work of adhesion. Asterisks indicate significant *P*-values. Work of adhesion decreased in wet conditions for all species except the terrestrial *A. pennsylvanica* and the riparian *W. clara*. The habitat×condition interaction term was significant; this may be because the loss in work of adhesion was smaller for species in the riparian habitat group and the riparian species *T. elongata* and *W. clara* showed very low values overall. Total sample size of discs tested, *N*=217.

**Fig. 6.**
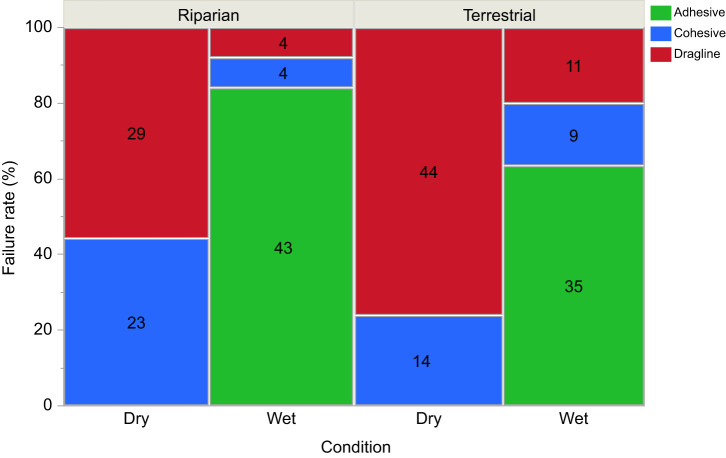
**Variation in failure mechanism of pyriform attachment discs.** The failure type shifted from predominantly draglines breaking in dry conditions to predominantly adhesive failure of the discs themselves in wet conditions for both habitat groups (*P*≤0.0001). The habitat×condition interaction term was not significant (*P*=0.75). Ambiguous disc failures (*n*=23) were coded as adhesive failure in this figure but coding them as cohesive failure gave identical statistical results. *N*=217.

The peak force of adhesion was significantly lower in wet loading conditions (*P*=<0.0001) and the habitat×condition interaction term was significant (*P*=0.0007) showing that the drop in force was greater for terrestrial species (Fig. 4). Pairwise comparisons showed that peak force of adhesion dropped for all terrestrial species in wet loading conditions but was not statistically different from that in dry loading conditions for the riparian species *T. elongata* and *W. clara.* Peak force of adhesion did decrease in wet loading conditions for the riparian species *D. triton*, but the decrease was about half that seen in terrestrial species (Fig. 4). The work of adhesion was also significantly lower in wet loading conditions (*P*≤0.0001), but both the habitat (*P*≤0.0001) and the habitat×condition interaction terms (*P*=0.003) were significant (Fig. 5). Overall, work of adhesion was higher for terrestrial species, but this likely reflects the very small size of *W. clara* discs. Only the terrestrial species *A. pennsylvanica* and the riparian species *W. clara* did not experience a significant decrease in the work of adhesion in wet conditions (Fig. 5). Failure mechanism changed from mostly draglines breaking in dry loading conditions to mostly adhesive failure in wet loading conditions for all species (Fig. 6). There was no difference between riparian and terrestrial species in failure mechanism (*P*=0.75) and coding of ambiguous observations did not change the statistical results, so Fig. 6 shows all ambiguous attachment disc failures coded as adhesive failure.

## DISCUSSION

We found that water reduced the strength and work of adhesion of pyriform silk attachment discs more for terrestrial species of spiders than for riparian species (Figs 4 and 5). The three riparian species in our study are phylogenetically distant from one another and likely reflect independent transitions between terrestrial and riparian lifestyles (Kulkarni et al., 2023). Therefore, our results support the hypothesis that natural selection has shaped the adhesive function of pyriform attachment discs in wet environments. A recent study examining the attachment discs of the fully aquatic spider species *Argyroneta aquatica* found that the adhesive performance of their attachment discs was not significantly impacted when the discs were spun underwater versus in dry conditions, further supporting the hypothesis that pyriform silk performance may evolve resistance to water during adaptation to aquatic/semi-aquatic lifestyles (Schaber et al., 2023).

*Dolomedes triton* is the only riparian species that showed a statistically significant decrease in peak force of adhesion during loading in wet conditions, albeit marginally (*P*=0.049). This drop may be due to sampling error or may reflect that *D. triton* is the only cursorial hunting species in our study, such that it may have experienced reduced selective pressure on the performance of attachment discs if this has less effect on prey capture success. *Agelenopsis pennsylvanica* was the only terrestrial species whose work of adhesion was not significantly decreased by wet conditions. This could reflect random variation among species or some difference in selective pressure relative to other terrestrial species, such as greater microhabitat exposure to water or the divergent architecture of the funnel webs constructed by *Agelenopsis* (Hopfe et al., 2024). A larger sample size of species across all web types is necessary to understand whether web architecture interacts with environmental conditions in the evolution of attachment disc function or whether the discrepancy we see simply reflects random chance. Regardless, when the species were grouped based on habitat, there was a significant difference between the terrestrial and riparian groups in the change in attachment disc performance from dry to wet conditions. We are confident that our species sampling encompasses three evolutionarily independent transitions between terrestrial and riparian lifestyles but cannot say with certainty whether the riparian microhabitats represent derived or ancestral lifestyles. Further studies should investigate the phylogenetic distribution of water-resistant attachment discs to identify whether this trait has convergently evolved in species as they enter riparian environments or whether it is an ancestral trait that terrestrial species shed when their niche no longer includes the adhesive constraint of wet environmental conditions.

The degree to which web or dragline performance in nature could be limited by reduced adhesion of wet pyriform attachment discs is unknown, but the resistance of riparian species pyriform discs to water may allow these species to better exploit niches in humid or wet areas. Terrestrial spider attachment discs typically support 2–7 times the body weight of the spider (Grawe et al., 2014). However, terrestrial species in this study lost about 50% of the maximum peak force of adhesion of their discs when wet. In dry conditions, many spiders' safety lines already operate close to the minimum threshold for surviving a fall without the safety line breaking (Ortlepp and Gosline, 2008). A 50% decrease in maximum force may result in safety lines that are unable to withstand the force of a falling spider without breaking. However, spiders gradually slow their fall as they abseil to stay well below the safety line breaking threshold (Brandwood, 1985). Personal observations verified that the structural webs of *L. hesperus* cobwebs do not spontaneously fail when the attachment discs are wetted. This may be due to several factors such as the synergistic effect of clusters of multiple attachment discs distributing stresses, variation in attachment disc adhesion on different substrates, or the passive tension of silk threads simply not being great enough to detach wet attachment discs.

Like a lot of adhesion research, this study was conducted using glass slides, a substrate that is significantly more hydrophobic and smoother than most substrates present in natural habitats. Attachment disc adhesion can decrease with increasing substrate hydrophobicity and smoothness, making the conditions used in this study more akin to a ‘worst-case scenario’ of adhesion for spiders (Wolff et al., 2020). Thus, the magnitude of decrease in performance of pyriform discs in wet conditions may not be as great for many natural substrates. Regardless, our study still provides insight into the adhesive properties of attachment discs when in a challenging environment.

Both terrestrial and riparian species showed a similar transition in failure mode from draglines breaking in dry conditions to adhesive failure of the pyriform discs in wet conditions. Thus, dragline tensile strength limits performance in dry conditions while the adhesive interaction between the attachment disc baseplate and the substrate limits performance in wet conditions. This suggests that the entire adhesive system is relatively well optimized, with similar safety factors in the different components. The short time frame that the attachment discs were exposed to water excludes the possibility of fouling reducing performance, and the lack of visible degradation or increased cohesive failure rates of the discs after exposure to water suggests that hydrolysis also has a minimal effect on performance. Because the discs were placed in dry conditions, the effects of water on spreading and curing should also be negligible. The shift to adhesive failure of pyriform discs for all species in wet conditions therefore suggests that interfacial bonding interference by water played the biggest role in our experiment. Notably, the peak force of adhesion for attachment discs was statistically indistinguishable between wet and dry conditions for two of the three riparian species tested, even though they showed the same dramatic increase in adhesive failure in wet conditions as the other spiders. This suggests that the performance of attachment discs spun by riparian species is more tolerant of adhesive failure. Riparian species' attachment discs could mitigate the effects of water on the peak force of adhesion through reduced reliance on hydrogen bonds to maintain adhesion. Alternatively, the discs may initially keep water molecules out of the interface through some compositional or morphological modification, until the discs were deformed enough from the tensile testing that water molecules penetrated into the interface to cause adhesive failure. Or, the microstructure of riparian species attachment discs may explain our data. Further studies are necessary to identify what mechanisms are taking place that cause the observed high peak forces while still failing adhesively.

Transitions in failure type also play a key role in controlling adhesion of viscid capture silk, which fails adhesively at lower humidity and shifts to cohesive failure at higher humidity (Amarpuri et al., 2015, 2017; Opell et al., 2018). Adhesive force is maximized near this transition point, although the humidity at which the transition occurs differs among species spinning webs in dryer versus wetter microhabitats. In our study, attachment discs showed an opposite pattern of transition from dragline failure in dry conditions to adhesive failure in wet conditions. This difference may be because attachment discs are initially placed as a liquid glue that spreads well regardless of environmental conditions and therefore makes good contact with the substrate before curing into a solid disc. A previous study found that *L. hesperus* attachment discs performed slightly better when spun onto a wet surface, possibly due to increased spreading of the glue before it cured (Steklis et al., 2025). Both viscid silk and pyriform silk performance responds to microhabitat humidity, but the differences in failure mechanism transitions might relate to the different ways these silks are used. Viscid silk needs to stay wet and maintain the potential to spread on surfaces for long periods of time until insect prey contact the glue droplets. In contrast, pyriform attachment discs just need to spread for a fraction of a second as the discs are first placed by the spider. Both viscid silk and attachment discs show evolutionary responses to adhesion in wet conditions, but the two silk types make use of different adhesive mechanisms because of the difference in functionality of the silks.

Our study adds to the growing body of evidence that environmental selective pressures shape the evolution of the functional properties of spider silks. For instance, species that live in unshaded environments produce viscid silk that is more resistant to the degrading effects of UV light (Stellwagen et al., 2015), and the atmospheric water uptake that optimizes glue viscosity for adhesion is specialized among species to match microhabitat differences in relative humidity (Amarpuri et al., 2015). Hopfe et al. (2024) also found that the material properties of the major ampullate silk frames of webs correlate with microhabitat differences among species in high rainfall versus dry environments. However, Wolff et al. (2020) found that pyriform discs adhered more poorly on hydrophobic substrates regardless of the common surface chemistry typical for species from different environments, concluding that there was no evidence of pyriform adaptation to the substrate surface polarity conditions of their microhabitats. Thus, our study provides some of the first evidence for microhabitat specialization of pyriform silk and suggests that specialization for environmental microhabitat conditions may be a general phenomenon for all types of silks produced by spiders.

We found that the adhesion of pyriform silk attachment discs is reduced significantly more by water for dry habitat- versus wet habitat-dwelling spider species. Riparian species' discs were more resistant to the effect of water on adhesion, even though the pyriform discs spun by all species shifted toward adhesive failure when wet. These findings provide some of the first evidence for ecological adaptation in pyriform silk and underscore the role of environmental pressure in shaping the evolution of spider silks. Our study may also help to inspire a more comparative approach to biomimetic investigations of biological adhesives produced by organisms such as amphibians, other invertebrates and plants. Future research that untangles the mechanistic differences in terrestrial and riparian spiders' attachment discs could inspire innovations in high-performance reversible adhesives that work in wet conditions, help refine models of polymer adhesion and broaden our understanding of the fundamental challenge of adhesion in wet environments.
